# Erythropoietin resistance-secondary to watermelon stomach: a forgotten story in chronic kidney disease patients

**DOI:** 10.11604/pamj.2023.44.40.36839

**Published:** 2023-01-20

**Authors:** Nikolaos Sabanis, Eleni Paschou

**Affiliations:** 1 Department of Nephrology, General Hospital of Trikala, Trikala, Greece; 2 Department of General Practice and Family Medicine, General State Hospital of Nikaia, Nikaia, Greece

**Keywords:** Gastric antral vascular ectasia, watermelon stomach, erythropoietin resistance, chronic kidney disease

## Image in medicine

Watermelon stomach, also known as gastric antral vascular ectasia (GAVE), is a rarely seen cause of acute upper gastrointestinal bleeding or chronic iron deficient anemia, revealed when gastroscopy is performed. GAVE affects predominantly women of advanced aged and is usually associated with certain underlying co-morbidities including liver cirrhosis, autoimmune diseases and chronic kidney disease. Based on the above, we report the case of a 75-year-old woman under hemodialysis for six consecutive years due to end-stage renal disease attributable to hypertensive nephrosclerosis. The patient also suffered from chronic ischemic heart disease, severe peripheral artery disease and an unidentified connective tissue-disease characterized by high levels of anti-nuclear antibodies and clinical symptoms restrained in flares of non-erosive polyarthritis. Six months prior, she developed hyporesponsiveness to recombinant human erythropoietin (rEPO) due to iron deficiency despite the administration of the appropriate dose of intravenous iron as well as the exclusion of other co-existing causes of resistance, namely inadequate hemodialysis, inflammation, malnutrition, severe hyperparathyroidism and underlying hematologic disorders. Surprisingly, the patient experienced a sudden episode of severe upper gastrointestinal bleeding characterized by hemodynamic instability, hemoglobin fall and melena that required blood transfusions. Once hemodynamic stabilization was achieved, an upper endoscopic exam was performed showing the pathognomonic extensive vascular ectasias and patchy erythematosus lesions at the distal antrum. Thus, GAVE should be considered in the differential diagnosis of chronic kidney disease patients who present not only with acute blood loss but also with resistance to rEPO due to inadequate iron stores.

**Figure 1:**
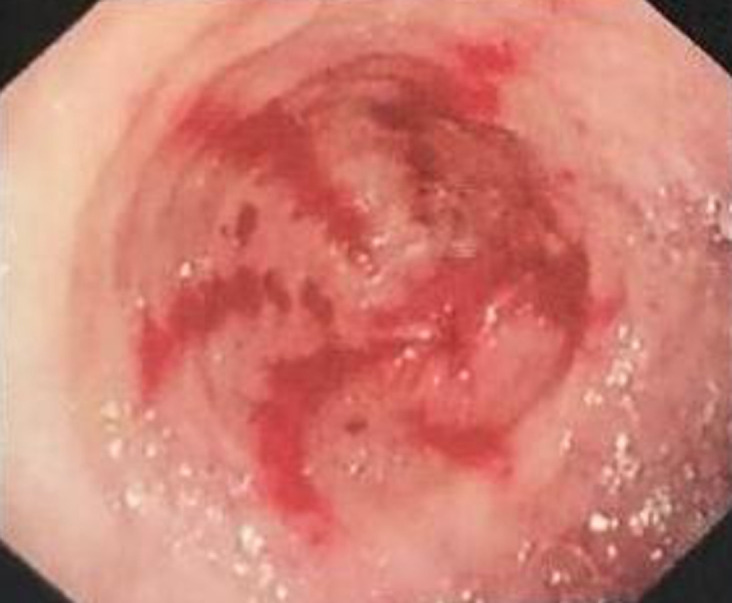
gastric antral vascular ectasia or watermelon stomach's endoscopic findings

